# Artificial Intelligence in Gastric Cancer: Diagnostic, Prognostic, and Predictive Developments, Evidence Maturity, and Translational Challenges

**DOI:** 10.1002/cnr2.70665

**Published:** 2026-10-08

**Authors:** Chong Chen, Weisong Xu, Tong Wu, Junpeng Zhao, Xuebing Xu, Mingbing Xiao, Kang Xu

**Affiliations:** ^1^ Department of Gastroenterology Affiliated Hospital and Medical School of Nantong University Nantong Jiangsu China; ^2^ Department of Gastroenterology Nantong University Affiliated Dongtai Hospital Yancheng Jiangsu China; ^3^ Department of Gastroenterology Nantong Rehabilitation Hospital Nantong City Jiangsu China

**Keywords:** artificial intelligence, clinical translation, computational pathology, deep learning, evidence appraisal, gastric cancer, radiomics, structured review

## Abstract

**Background:**

Gastric cancer (GC) remains a major global health burden characterized by substantial heterogeneity in diagnosis, prognosis, and treatment response. Artificial intelligence (AI), including machine learning (ML) and deep learning (DL), has been increasingly investigated for the analysis of endoscopic images, radiological scans, whole‐slide pathology images, molecular profiles, liquid‐biopsy data, and multimodal clinical datasets. This structured review summarizes recent AI‐based developments in GC diagnosis, staging, prognostic assessment, and treatment‐response prediction, with particular emphasis on the translational maturity of the available evidence. PubMed/MEDLINE, Web of Science, Embase, Scopus, and Google Scholar were searched for relevant English‐language publications from database inception through January 2026. Eligible studies were evaluated according to clinical task, data modality, model type, cohort size, study design, validation strategy, interpretability, clinical workflow readiness, and reported limitations.

**Recent Findings:**

Recent AI models have demonstrated promising performance in selected research settings, particularly in endoscopic image analysis, computational pathology, CT‐based radiomics, and multimodal prognostic modeling. However, most studies remain retrospective, single‐center, and internally validated. Calibration, robustness, prospective clinical utility, and generalizability across populations and institutions remain insufficiently assessed. Major barriers to translation include data heterogeneity, annotation variability, overfitting, data leakage, external validation failure, algorithmic bias, regulatory requirements, reimbursement, clinician‐AI interaction, medicolegal responsibility, post‐deployment monitoring, and model drift.

**Conclusion:**

AI has considerable potential to support GC diagnosis, prognosis, and treatment planning, but most current models remain insufficiently validated for routine clinical use. Future progress will depend on transparent reporting, standardized validation, multicenter prospective trials, clinically meaningful endpoints, and the integration of explainable, trustworthy AI systems into routine clinical workflows.

## 1 Introduction

Gastric cancer (GC) remains a major global cancer burden. According to GLOBOCAN 2022 estimates, stomach cancer accounted for 968 784 new cases and 660 175 deaths worldwide, ranking fifth for both incidence and cancer‐related mortality [1]. This epidemiological burden is unevenly distributed, with higher incidence in East Asia and other high‐risk regions, and is shaped by environmental exposures, dietary factors, endemic *Helicobacter pylori* infection, and regional differences in screening and treatment pathways [1, 2, 3].

A major clinical challenge is that many patients are diagnosed after progression beyond early‐stage disease, when curative treatment is more difficult and long‐term outcomes are poorer [2, 3]. Early diagnosis through esophagogastroduodenoscopy (EGD) with biopsy and histopathological confirmation can markedly improve prognosis, whereas advanced‐stage GC is associated with substantially lower survival [2, 3]. At the same time, GC is characterized by marked clinical, histological, and molecular heterogeneity, including genomically stable, chromosomal instability, microsatellite instability, Epstein–Barr virus‐associated, and additional transcriptome‐defined subtypes [4, 5]. These biologically distinct subgroups differ in tumor behavior, prognosis, immune microenvironment, and therapeutic vulnerability, complicating risk stratification and treatment selection [4, 5].

Current diagnostic and therapeutic approaches for GC therefore have important limitations. Endoscopic detection of subtle early lesions and manual histopathological evaluation remain influenced by operator experience, image quality, sampling adequacy, interobserver variability, and workload [2, 3]. Conventional imaging has limited ability to non‐invasively assess lymph node metastasis, lymphovascular invasion, molecular phenotypes, and recurrence risk [2, 3]. In systemic therapy, trastuzumab, immune checkpoint inhibitors, and other biomarker‐directed strategies have improved outcomes only in selected molecularly defined subgroups, while current biomarkers do not fully capture the complexity of treatment response [6, 7, 8].

These diagnostic and therapeutic gaps have motivated increasing investigation of artificial intelligence (AI), including machine learning (ML) and deep learning (DL), across endoscopic images, computed tomography and PET imaging, whole‐slide pathology images, liquid biopsy assays, microbiome profiles, transcriptomic datasets, and multimodal clinical records [9, 10, 11, 12, 13, 14, 15, 16, 17, 18, 19, 20, 21, 22, 23, 24]. By learning high‐dimensional patterns, AI models may support lesion detection, staging, biomarker inference, recurrence prediction, treatment‐response modeling, and workflow triage; however, the majority of published GC models remain at the development or early validation stage rather than established clinical tools [9, 10, 11, 12, 13, 14, 15, 16, 17, 18, 19, 20, 21, 22, 23, 24]. Reported performance metrics should therefore be interpreted in relation to study design, cohort diversity, validation strategy, calibration, decision‐curve analysis, risk of bias, and clinical workflow readiness [9].

The objective of this structured review is to synthesize recent AI‐based developments in GC while explicitly distinguishing research‐stage model development from evidence supporting clinical translation. Unlike prior reviews that primarily summarize model performance across diagnostic or prognostic tasks, we evaluate the literature through a translational evidence framework that considers validation hierarchy, study design, reproducibility, interpretability, workflow readiness, and implementation barriers [9]. To avoid repeating earlier model performance catalogues, this review emphasizes citation accuracy, methodological limitations, comparative evidence maturity, and implementation requirements. This perspective is intended to clarify which AI applications currently have comparatively stronger evidence, which remain exploratory, and what steps are required before routine clinical adoption can be justified.

## 2 Methods

This article was revised as a structured narrative review with a scoping approach. We searched PubMed/MEDLINE, Web of Science, Embase, Scopus, and Google Scholar for English‐language studies from database inception through January 2026, with the final literature update conducted during manuscript revision. Searches addressed AI, ML, DL, radiomics, computational pathology, endoscopy‐based AI, liquid biopsy, multi‐omics, prognosis, or treatment‐response prediction in gastric cancer. The search combined terms related to gastric cancer or stomach cancer with terms related to artificial intelligence, machine learning, deep learning, neural networks, radiomics, pathomics, endoscopy, computational pathology, multimodal prediction, prognosis, recurrence, chemotherapy, immunotherapy, targeted therapy, and clinical translation.

Eligible publications included original studies, clinically oriented methodological studies, and high‐quality reviews where necessary for background or conceptual context. Titles/abstracts and full texts were screened for topical relevance, accessibility of methodological detail, and relevance to clinically meaningful gastric cancer tasks. Primary gastric cancer studies were prioritized for claims regarding model performance, diagnostic accuracy, prognostic stratification, or treatment‐response prediction. Studies of other malignancies were excluded from gastric cancer‐specific claims unless explicitly presented as cross‐tumor or hypothesis‐generating evidence. We also excluded articles without accessible methodological detail, studies with insufficient relevance to gastric cancer, and secondary reviews used to support primary biological or clinical claims when original clinical trials or molecular studies were more appropriate.

Data extraction focused on clinical task, data modality, model type, cohort size, study design, number of centers, geographic setting, validation strategy, endpoint definition, reported discrimination metrics, calibration or decision‐curve analysis when available, explainability method, and translational status. Because this was a structured narrative/scoping review rather than a quantitative systematic review, we did not assign pooled effect estimates or perform a meta‐analysis; this decision reflected substantial heterogeneity across modalities, endpoints, model architectures, and reporting standards. Instead, methodological quality and translational readiness were appraised qualitatively using the evidence framework described below.

## 3 Conceptual Framework for Evidence Appraisal

To address the risk of overinterpreting development‐stage models, we evaluated the literature using a translational evidence framework. Studies were considered to have lower translational maturity when they relied on retrospective single‐center data, internal split‐sample validation only, small or highly selected cohorts, unclear annotation procedures, class imbalance, absence of calibration analysis, or lack of external testing. Higher translational maturity was assigned to studies with multicenter cohorts, independent external validation, prespecified endpoints, prospective evaluation, reader or workflow studies, transparent reporting, explainability, and evidence of clinical utility beyond AUC or accuracy.

We also considered model‐family suitability. CNNs are useful for local image‐pattern recognition in endoscopy, CT, and pathology but may be sensitive to domain shift. Transformer‐based models can capture broader spatial or contextual relationships but usually require larger datasets and greater computational resources. Multiple instance learning is well suited to weakly supervised whole‐slide pathology analysis but may provide limited instance‐level interpretability. Graph neural networks can model molecular or spatial relationships but depend heavily on graph construction assumptions. Radiomics‐based ML models may be more transparent but are sensitive to imaging protocols and feature reproducibility. Self‐supervised learning, foundation models, vision‐language models, weak supervision, and large multimodal systems are promising directions, but gastric cancer‐specific validation remains limited.

## 4 AI in the Diagnosis and Staging of Gastric Cancer

AI models have been investigated as tools to support GC diagnosis and staging by analyzing complex endoscopic, radiologic, and pathological data (Figure 1). However, most evidence remains derived from retrospective model‐development studies, and performance should be interpreted according to validation design, cohort representativeness, calibration, and clinical workflow readiness rather than discrimination metrics alone.

**FIGURE 1:**
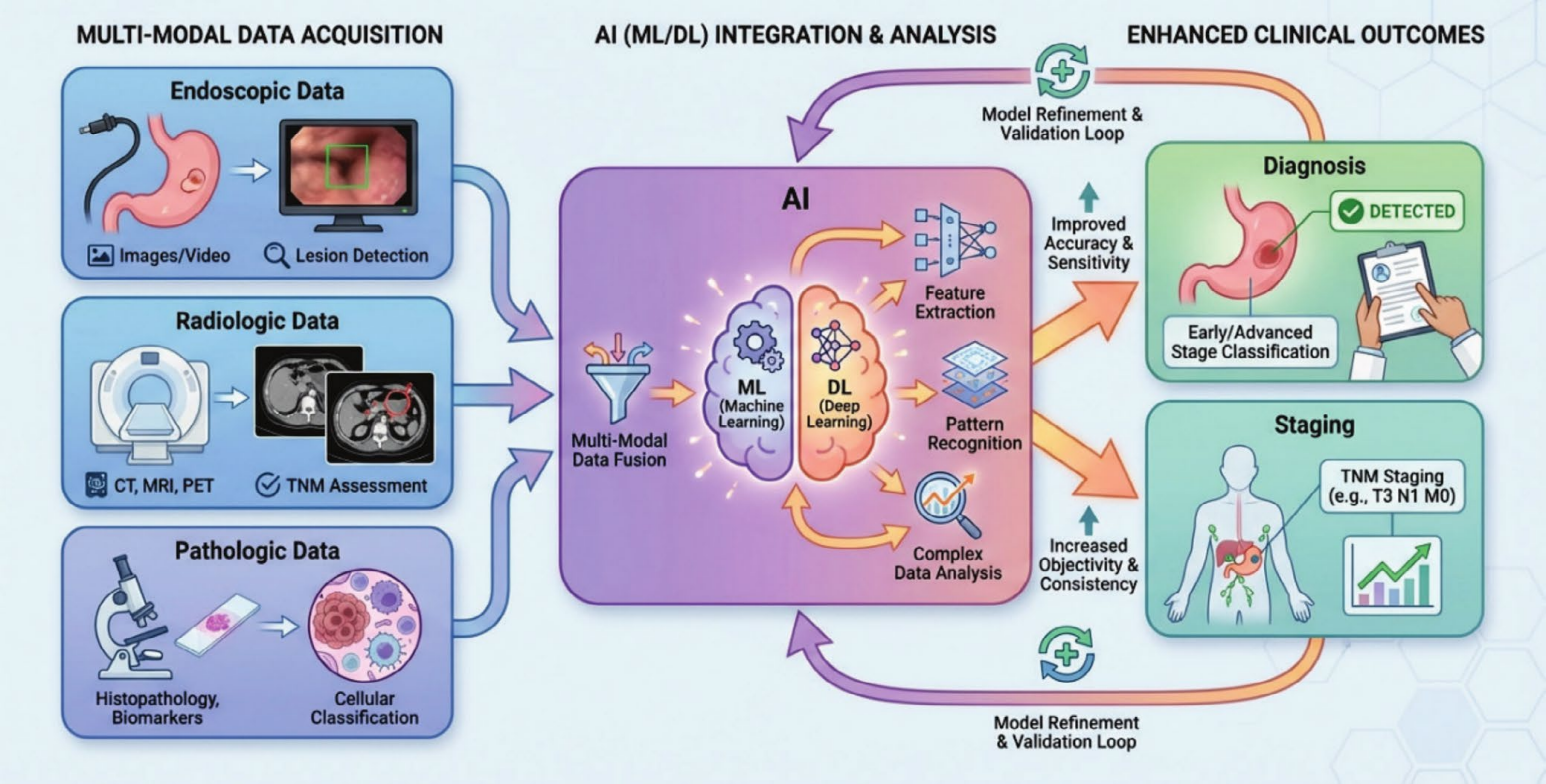
Overview of AI‐driven multi‐modal data integration for the diagnosis and staging of gastric cancer. Artificial intelligence, encompassing machine learning (ML) and deep learning (DL), can process high‐dimensional data from endoscopic, radiologic, and histopathologic sources to support diagnostic assessment and staging in research or decision‐support contexts. *Source:* Created by the authors based on the reviewed literature; no previously published figure was reused.

### 4.1 AI in Histopathological Analysis

Computational pathology is an active area of AI research for GC diagnosis, biomarker inference, and risk stratification. Whole‐slide images (WSIs) provide rich morphologic information, but their gigapixel scale, staining variability, scanner differences, annotation burden, and case‐mix heterogeneity create substantial methodological challenges. Patch‐based pipelines and multiple instance learning (MIL) are commonly used to process WSIs, but their outputs require external validation and careful interpretability assessment before clinical implementation. Representative AI applications in gastric cancer histopathological analysis are summarized in Table 1.

**TABLE 1:** Summary of AI applications in gastric cancer histopathological analysis.

AI model/method	Application/function	References	Evidence appraisal/limitations
Deep learning (DL) model	Staged gastric cancer (GC) from whole slide images (WSIs) with 100% accuracy and 0.999 AUC.	[10]	Balanced binary dataset; no independent external validation; interpret near‐perfect metrics cautiously; possible overfitting/methodological inflation.
Interpretable DL model	Predicted dMMR/MSI‐H status from H&E slides (AUCs up to 0.930); XAI features improved pathologist accuracy.	[11]	Research‐stage evidence; external validation, calibration, decision‐curve analysis, and workflow utility remain limited unless explicitly reported.
DL model	Predicted Epstein–Barr Virus (EBV) status from biopsies (AUC 0.87) to screen for EBER‐ISH testing.	[12]	Research‐stage evidence; external validation, calibration, decision‐curve analysis, and workflow utility remain limited unless explicitly reported.
DL model	Subtyped GC based on Laurén classification for prognostication.	[13]	Research‐stage evidence; external validation, calibration, decision‐curve analysis, and workflow utility remain limited unless explicitly reported.
DL model	Derived prognostic risk scores from IHC data, outperforming TNM staging.	[14]	Research‐stage evidence; external validation, calibration, decision‐curve analysis, and workflow utility remain limited unless explicitly reported.
DL model	Analyzed cancer‐surrounding tissue to improve diagnosis from limited biopsies (93.96% accuracy).	[15]	Research‐stage evidence; external validation, calibration, decision‐curve analysis, and workflow utility remain limited unless explicitly reported.
Multiple instance learning (MIL)	A patch‐based method for processing large WSIs.	[14]	Research‐stage evidence; external validation, calibration, decision‐curve analysis, and workflow utility remain limited unless explicitly reported.
Hybrid CNN‐transformer model	Combined local and global analysis for GC classification (99.2% accuracy) with Grad‐CAM for interpretability.	[16]	Research‐stage evidence; external validation, calibration, decision‐curve analysis, and workflow utility remain limited unless explicitly reported.

Several DL models have reported high accuracy for tumor classification or staging from WSIs. For example, one model reported 100% accuracy and 0.999 AUC for binary stage classification [10]. These near‐perfect metrics should be interpreted cautiously because the study used a limited, perfectly balanced dataset and did not include an independent external validation cohort; therefore, the results may reflect overfitting, dataset‐specific features, or methodological inflation rather than real‐world diagnostic performance.

Beyond morphological classification, AI models are being investigated for predicting molecular phenotypes from routine hematoxylin and eosin (H&E) slides. Interpretable DL models have reported promising performance for dMMR/MSI‐H prediction, and EBV‐status prediction from biopsy specimens has also been explored. However, these models should be considered screening or triage tools pending prospective validation against established molecular assays and evaluation of performance across institutions, scanners, staining protocols, and patient populations.

For prognostic stratification, WSI‐based models may assist in Laurén classification and identification of morphologic patterns associated with outcome. Hybrid CNN‐Transformer and MIL‐based approaches are technically attractive because they combine local cellular features with broader tissue architecture. Nevertheless, evidence of clinical utility remains limited unless models demonstrate reproducibility, calibration, explainability, and incremental value over pathologist assessment and established clinicopathologic variables.

### 4.2 AI in Radiologic Image Analysis

Radiomics, defined as the high‐throughput extraction of quantitative spatial and textural features from images, enables non‐invasive profiling of tumor macro‐ and microenvironmental features using radiological data [17]. In GC, CT‐ and PET‐based AI models have been investigated for preoperative staging and prediction of aggressive pathological features (Figure 2), but feature reproducibility, scanner variability, segmentation protocols, and external validation remain central concerns. Representative radiologic AI models for GC staging are summarized in Table 2.

**FIGURE 2:**
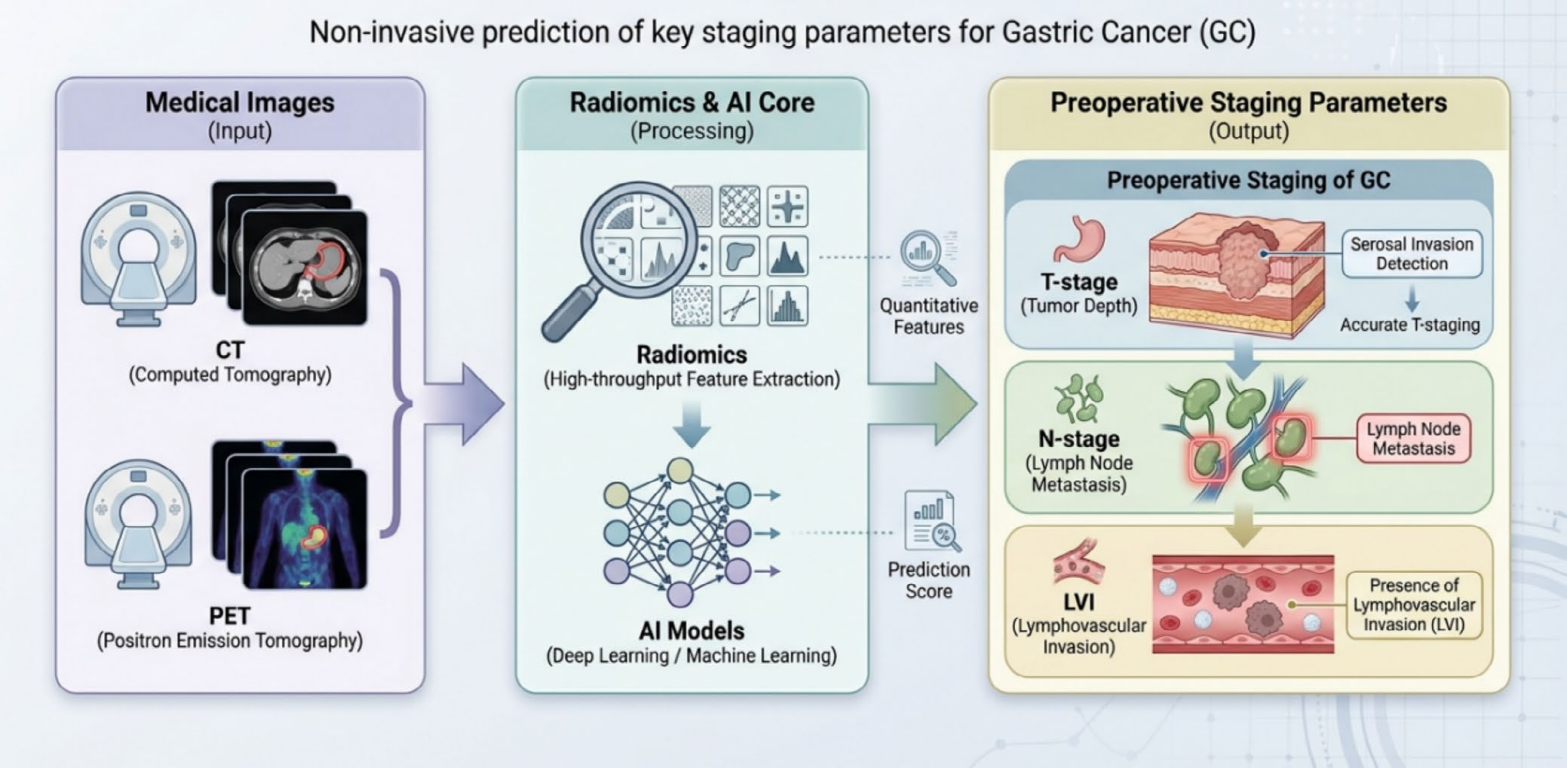
Radiomics and AI‐driven workflows in radiologic image analysis. High‐throughput quantitative features extracted from computed tomography (CT) and positron emission tomography (PET) can be analyzed by AI algorithms to estimate preoperative staging parameters, including T‐stage, N‐stage, and lymphovascular invasion. *Source:* Created by the authors based on the reviewed literature; no previously published figure was reused.

**TABLE 2:** Summary of AI models in radiologic image analysis for GC staging.

Name/approach	Application/function	References	Evidence appraisal/limitations
T‐staging (serosal invasion)	A logistic regression model integrating dual‐energy CT (DECT) parameters (iodine concentration, monochromatic attenuations) to predict serosal invasion and correlate with disease‐free survival.	[18]	Research‐stage evidence; external validation, calibration, decision‐curve analysis, and workflow utility remain limited unless explicitly reported.
N‐staging (LNM in lesser curvature)	A hybrid radiomics model combining features from the primary tumor and the entire lesser curvature on dual‐phase contrast‐enhanced CT (CECT) to predict lymph node metastasis.	[19]	Research‐stage evidence; external validation, calibration, decision‐curve analysis, and workflow utility remain limited unless explicitly reported.
N‐staging (LNM in non‐enlarged nodes)	A model combining the Node Reporting and Data System (Node‐RADS) with CT radiomics to improve metastasis prediction in non‐enlarged lymph nodes.	[20]	Research‐stage evidence; external validation, calibration, decision‐curve analysis, and workflow utility remain limited unless explicitly reported.
N‐staging (individual LNM)	A model combining CT features (enhancement pattern, diameter) with the Ki67 pathological marker to predict metastasis in individual lymph nodes using GEE logistic regression.	[21]	Research‐stage evidence; external validation, calibration, decision‐curve analysis, and workflow utility remain limited unless explicitly reported.
N‐staging (LNM risk)	A deep learning radiomics model based on dual‐energy CT (DECT) to improve the prediction of lymph node metastasis risk.	[22]	Research‐stage evidence; external validation, calibration, decision‐curve analysis, and workflow utility remain limited unless explicitly reported.
LVI prediction	A 2.5D deep learning model analyzing the tumor's maximum cross‐section and adjacent slices from venous phase CECT to predict lymphovascular invasion.	[23]	Research‐stage evidence; external validation, calibration, decision‐curve analysis, and workflow utility remain limited unless explicitly reported.

#### 4.2.1 T‐Staging and Serosal Invasion

Accurate T‐staging, specifically detection of serosal invasion (SI, T4a stage), is clinically important because SI is associated with peritoneal metastasis risk. Dual‐energy CT (DECT)‐derived functional parameters, combined with conventional clinical modeling, have reported improved SI prediction compared with conventional imaging in multicenter validation (AUC: 0.837–0.860) and an association with postoperative disease‐free survival [18]. These findings support further evaluation but do not by themselves establish routine clinical implementation.

#### 4.2.2 N‐Staging (Lymph Node Metastasis)

Predicting LNM in radiologically non‐enlarged nodes remains a persistent clinical dilemma. Hybrid radiomics models integrating features from the primary tumor and regional anatomy (e.g., the lesser curvature) on dual‐phase contrast‐enhanced CT (CECT) have reported improved discrimination compared with single‐region analyses [19]. Models incorporating the standardized Node Reporting and Data System (Node‐RADS) with CT radiomics have also been proposed to improve detection in borderline nodes [20]. Multimodal generalized estimating equation (GEE) approaches combining CT enhancement patterns with pathological markers such as Ki67 have shown promise for individual‐node metastasis prediction, but their clinical role requires prospective workflow evaluation [21]. Dual‐energy CT‐based deep learning radiomics models have also been proposed to improve lymph node metastasis risk prediction [22].

#### 4.2.3 Predicting Lymphovascular Invasion (LVI)

LVI is a clinically important marker of recurrence risk. AI facilitates preoperative LVI prediction through CT radiomics, 2.5D/3D DL algorithms, and PET/CT‐based models. In this review, gastric cancer‐specific evidence is emphasized for GC claims, whereas studies in esophageal or colorectal cancer are considered cross‐tumor evidence only and should not be used to support GC‐specific performance estimates.

### 4.3 AI in Endoscopic Analysis

Esophagogastroduodenoscopy (EGD) with biopsy and histopathological confirmation remains the diagnostic gold standard for GC. However, detection of early gastric cancer (EGC)‐which may present as subtle mucosal discoloration, minimal elevation or depression, or irregular microvascular patterns‐is highly operator‐dependent [24]. The AI‐augmented endoscopic workflow, including lesion detection, qualitative assessment, and estimation of invasion depth or tumor margins, is illustrated in Figure 3. Representative AI systems used in endoscopic analysis are summarized in Table 3.

**FIGURE 3:**
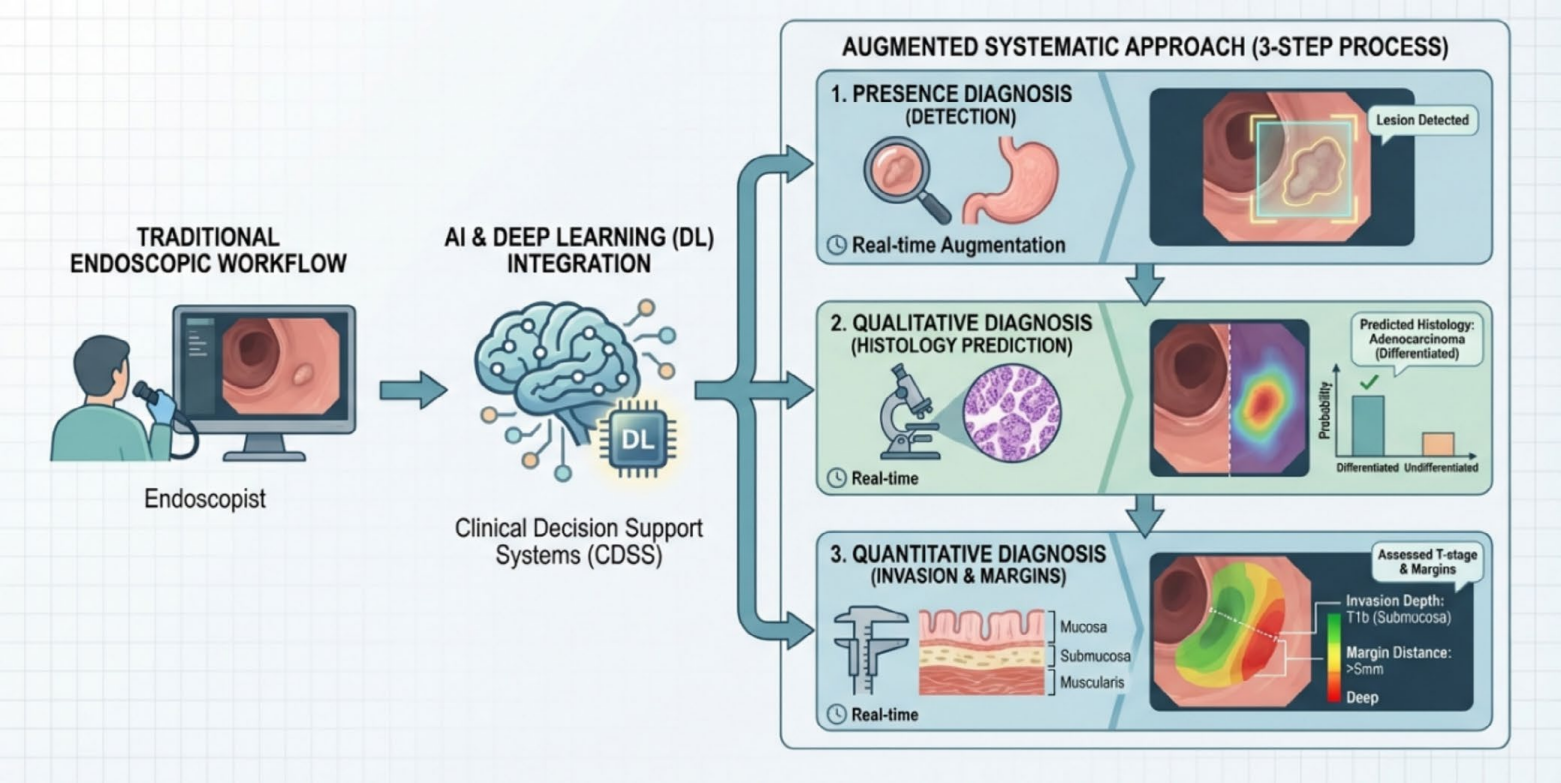
AI‐augmented systematic approach in endoscopic analysis. Deep learning‐based clinical decision support systems (CDSS) may assist the traditional endoscopic workflow through lesion detection, qualitative assessment, and estimation of invasion depth or tumor margins. *Source:* Created by the authors based on the reviewed literature; no previously published figure was reused.

**TABLE 3:** Summary of AI systems in endoscopic analysis.

Name/approach	Application/function	References	Evidence appraisal/limitations
General endoscopic AI	Assists in the systematic three‐step diagnostic process: presence, qualitative, and quantitative diagnosis.	[25]	Research‐stage evidence; external validation, calibration, decision‐curve analysis, and workflow utility remain limited unless explicitly reported.
General endoscopic AI	Improves the detection of early gastric cancer (EGC) by identifying subtle mucosal changes.	[24]	Research‐stage evidence; external validation, calibration, decision‐curve analysis, and workflow utility remain limited unless explicitly reported.
DL‐based CDSS	A system for automated, real‐time detection and classification of gastric neoplasms, including prediction of histopathology and invasion depth.	[26]	Research‐stage evidence; external validation, calibration, decision‐curve analysis, and workflow utility remain limited unless explicitly reported.
Real‐time video DL system	Reported performance comparable to or better than expert endoscopists in selected settings in detecting neoplasms and predicting tumor characteristics.	[27]	Research‐stage evidence; external validation, calibration, decision‐curve analysis, and workflow utility remain limited unless explicitly reported.
ENDOANGEL‐LA (XAI system)	A hybrid “logical anthropomorphic” system that mimics expert reasoning by integrating endoscopic features with DL/ML classifiers.	[28]	Case–control/development‐validation setting with expert comparison; requires magnification endoscopy and resource‐dependent deployment evaluation.
Static image‐based model	Performance significantly decreased when applied to video clips (accuracy dropped from 82.7% to 56.6%), highlighting the limitation of static analysis.	[29]	Research‐stage evidence; external validation, calibration, decision‐curve analysis, and workflow utility remain limited unless explicitly reported.
Purpose‐built video classifier	Maintained high accuracy (83.7%) on video analysis by incorporating sequential data, producing more stable predictions than static models.	[29]	Research‐stage evidence; external validation, calibration, decision‐curve analysis, and workflow utility remain limited unless explicitly reported.

In prospective settings, DL systems analyzing real‐time videos have achieved diagnostic performance comparable to or exceeding that of expert endoscopists [27]. Recognizing that static images lack important dynamic spatio‐temporal information (e.g., peristalsis, lesion hardness), modern AI systems prioritize video‐based analysis. Static models experience severe performance drops (e.g., 82.7% to 56.6%) when applied to videos, whereas purpose‐built sequential video classifiers maintain high, stable accuracy [29].

To foster clinical trust and mitigate the “black box” limitation, hybrid logical anthropomorphic XAI systems (e.g., ENDOANGEL‐LA) integrate established endoscopic visual criteria such as demarcation lines and microstructure patterns with ML classifiers [28]. The cited study reported 89% accuracy for expert endoscopists and 88.76% accuracy for ENDOANGEL‐LA; however, this system requires magnification endoscopy, which may limit deployment in lower‐resource settings [28, 30].

### 4.4 AI in Emerging Modalities

AI‐related GC diagnostic research also extends to emerging biological matrices, including liquid biopsies, the microbiome, and advanced molecular imaging. Preoperative assessment of lymph node metastasis in early T1 GC is clinically important because it informs the decision between endoscopic resection and radical gastrectomy. An AI‐analyzed epigenetic liquid‐biopsy assay using cell‐free DNA methylation markers has been developed to identify lymph node metastasis preoperatively (AUC: 0.86) [31]. An SVM‐based stool DNA methylation test using a four‐gene panel reported high specificity (97.8%) but more modest sensitivity (67.5%); in a screening context, this sensitivity implies that a substantial proportion of true positive cases may be missed and must be reported alongside specificity [32] (Figure 4).

**FIGURE 4:**
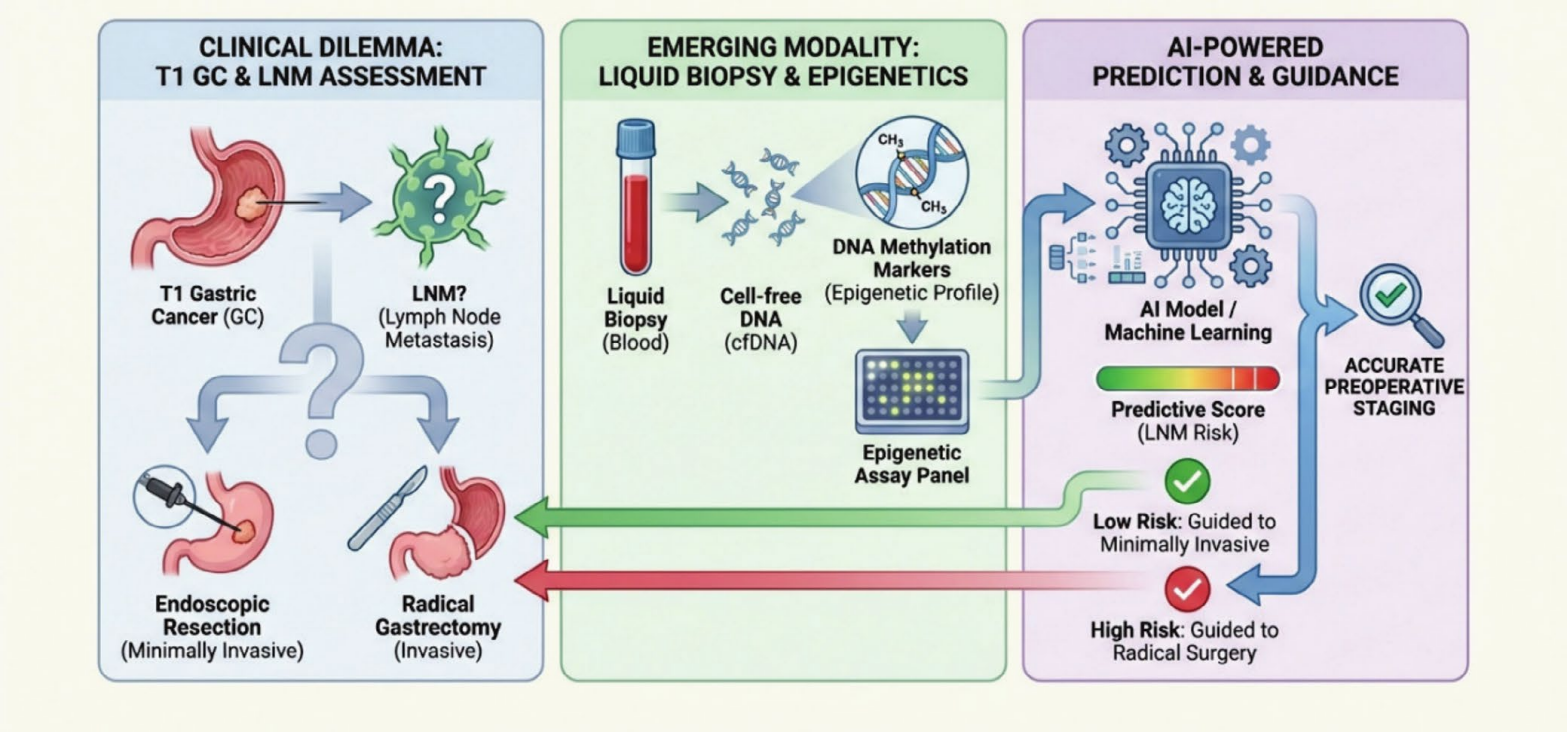
AI applications in emerging diagnostic modalities and liquid biopsies. AI‐powered predictive models can use epigenetic data, such as cell‐free DNA (cfDNA) methylation markers, to estimate the preoperative risk of lymph node metastasis in early (T1) gastric cancer and may inform selection between endoscopic resection and radical surgery. *Source:* Created by the authors based on the reviewed literature; no previously published figure was reused.

High‐throughput omics data may benefit from ML pattern recognition, but evidence remains preliminary. Serum N‐glycome signatures, microbiome‐derived extracellular vesicles, and novel PET tracers are promising research areas; however, many studies require larger validation cohorts, standardized pre‐analytic procedures, and comparison with existing diagnostic pathways before clinical utility can be inferred [33, 34, 35]. Representative AI applications in emerging diagnostic and staging modalities are summarized in Table 4.

**TABLE 4:** AI applications in emerging diagnostic and staging modalities.

Name/approach	Application/function	References	Evidence appraisal/limitations
cfDNA methylation assay	A liquid biopsy assay using a panel of cell‐free DNA methylation markers to preoperatively diagnose lymph node metastasis in T1 gastric cancer (GC).	[31]	Research‐stage evidence; external validation, calibration, decision‐curve analysis, and workflow utility remain limited unless explicitly reported.
Stool DNA methylation test	SVM‐based four‐gene stool DNA methylation panel for early GC detection; reported specificity 97.8% and sensitivity 67.5%.	[32]	Screening‐oriented biomarker study; sensitivity indicates false‐negative risk, and specificity alone is insufficient for screening utility.
Serum N‐glycomics model	A random forest model using a panel of 22 serum N‐glycans to distinguish GC patients from healthy controls with high accuracy (AUC > 0.90).	[33]	Research‐stage evidence; external validation, calibration, decision‐curve analysis, and workflow utility remain limited unless explicitly reported.
EV metagenome profiling	A logistic regression model using 16S rRNA profiles of bacteria‐derived extracellular vesicles (EVs) for GC detection; urine EVs showed the highest performance.	[34]	Research‐stage evidence; external validation, calibration, decision‐curve analysis, and workflow utility remain limited unless explicitly reported.
Dual‐target PET tracer (18F‐FAPI‐42‐RGD)	An engineered PET tracer that simultaneously targets FAP and integrin αvβ3, showing increased tumor uptake in preclinical GC models compared to single‐target tracers.	[35]	Primarily molecular‐imaging/preclinical evidence; not yet a validated clinical AI model for GC decision‐making.

## 5 AI for Prognosis and Risk Stratification

AI‐based prognostic models have shown potential to refine risk stratification in GC research settings, but their clinical impact remains uncertain because many models have not undergone prospective validation or workflow evaluation. By integrating high‐dimensional data, AI algorithms may identify prognostic patterns not captured by conventional clinicopathological variables; however, model discrimination alone is insufficient to establish clinical usefulness (Figure 5). Representative AI models for prognosis and risk stratification are summarized in Table 5.

**FIGURE 5:**
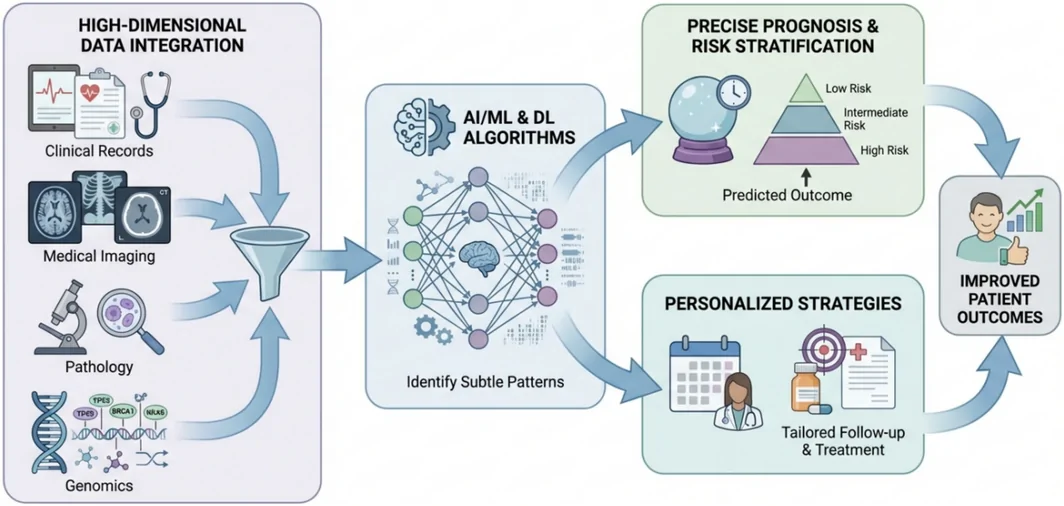
Multimodal AI frameworks for prognosis and risk stratification. By integrating high‐dimensional data from clinical records, medical imaging, computational pathology, and multi‐omics, AI algorithms may identify prognostic patterns that support risk stratification and follow‐up planning after appropriate validation. *Source:* Created by the authors based on the reviewed literature; no previously published figure was reused.

**TABLE 5:** Summary of AI models for prognosis and risk stratification.

Name/approach	Application/function	References	Evidence appraisal/limitations
Random forest (RF) model	Predicted postoperative complications in gastric and colorectal cancer. Performed well among eight ML models. Key predictors in gastric cancer were BMI, number of examined lymph nodes, and age. An application‐based tool was developed using SHAP for interpretability.	[36]	Prospective nationwide multicenter cohort; promising for complication prediction, but clinical workflow impact still needs evaluation.
ML models and dynamic nomogram	Six ML models (including RF, XGBoost, ANN) were developed to predict complications after radical gastrectomy. A simpler, interpretable dynamic nomogram based on six clinical risk factors achieved performance (AUC = 0.856) comparable to the best‐performing RF model.	[37]	Research‐stage evidence; external validation, calibration, decision‐curve analysis, and workflow utility remain limited unless explicitly reported.
Nomograms (general application)	A key data visualization tool for risk assessment, typically built from multi‐factor regression models. Numerous nomograms have been developed to predict specific postoperative complications like intra‐abdominal infections, anastomotic leakage, and deep vein thrombosis.	[38]	Research‐stage evidence; external validation, calibration, decision‐curve analysis, and workflow utility remain limited unless explicitly reported.

### 5.1 Prediction of Postoperative Complications

Surgical complications can adversely affect recovery and long‐term outcomes. ML models can capture non‐linear relationships among preoperative variables that may be useful for complication prediction. In a prospective multicenter study of 3926 patients (PACAGE database), Random Forest (RF) algorithms performed well for overall and infectious complications, identifying BMI, age, and examined lymph node count as important risk factors [36].

To bridge the gap between complex algorithms and clinical utility, researchers frequently use SHAP (Shapley Additive exPlanations) values and interactive web tools. A simpler dynamic nomogram built through ML feature selection (incorporating age, BMI, ASA grade, and related variables) has achieved discrimination comparable to more complex models (AUC = 0.856) in one study [37]. Data‐driven feature selection has also suggested that preoperative complete‐blood‐count parameters and 3D‐CNN‐derived visceral adipose‐tissue radiomics may contribute to early surgical morbidity prediction [39, 40]. Time‐sequential ML models can incorporate perioperative data to update complication risk across short postoperative intervals [41].

### 5.2 Multimodal Data Fusion for Long‐Term Survival and Recurrence

Predicting long‐term outcomes such as overall survival (OS) and recurrence increasingly relies on multimodal data fusion. One interpretable Risk Stratification Assessment (RSA) model integrated clinical variables, CT radiomics, and WSI pathomics and reported an AUC of 0.903 for early recurrence prediction [42]. Although promising, this result should not be characterized as definitive or landmark evidence without independent prospective validation and demonstration of incremental clinical value over established staging and treatment variables.

DL architectures such as “MultiDeepsurv” use hybrid CNN‐Transformer networks to extract pathology features and Graph Convolutional Networks (GCNs) to model genomic relationships, generating prognostic signatures that have reported performance beyond traditional TNM staging in retrospective analyses [43]. Similarly, Random Survival Forest approaches incorporating environmental and host genetic data (e.g., single nucleotide polymorphisms) have been proposed to refine survival prediction [44].

Dynamic prediction models extend baseline risk estimation by updating recurrence risks longitudinally. A model incorporating time‐dependent changes in the Prognostic Nutritional Index (PNI) during postoperative follow‐up reported improved prediction of three‐year recurrence compared with static models [45]. Standard clinical variables and serum markers (AFP, CEA, CA125, and CA19‐9) have also been incorporated into nomograms for 3‐year survival stratification [46, 47]. AI‐enabled analyses of operational timelines have additionally generated hypotheses about surgical triage, including the observation that longer wait times may not inherently worsen mortality for selected early‐stage patients [48].

### 5.3 Molecular Signatures and Radiogenomics for Prognostication

Radiogenomics links macroscopic imaging phenotypes to underlying gene‐expression patterns. Using weighted gene co‐expression network analysis (WGCNA) combined with CT radiomics, researchers have identified imaging biomarkers that have reported improved OS prediction compared with TNM staging in development or retrospective validation settings [49, 50].

At the molecular level, ML algorithms such as LASSO‐Cox and SVM‐RFE have been used to mine transcriptomic datasets and construct prognostic gene signatures. Signatures derived from cancer‐associated adipocytes, THY1, RAD51, and THSD7A have been reported to stratify patients in retrospective or public‐database analyses [51, 52, 53, 54]. These findings should be interpreted as hypothesis‐generating until they demonstrate reproducibility across independent clinical cohorts and prospective utility.

### 5.4 Preoperative Prediction of High‐Risk Tumor Characteristics

Effective risk stratification requires early identification of aggressive tumor characteristics such as LVI and distant metastasis. Gastric cancer‐specific CT and PET/CT radiomics studies have reported promising performance for preoperative LVI prediction [55, 56, 57]. Studies in esophageal squamous cell carcinoma or colorectal cancer should not be used as direct evidence for GC‐specific LVI prediction; if discussed, they should be explicitly labeled as cross‐tumor methodological evidence rather than gastric cancer evidence.

Similarly, integrating molecular risk scores with clinical variables has been explored for predicting peritoneal metastasis and other high‐risk features [58, 59]. AI‐based analyses of large databases can generate clinically relevant hypotheses, such as the prognostic role of tumor size in T1 GC [60], but such results should not be described as reshaping staging paradigms unless independently confirmed and incorporated into clinical guidelines.

### 5.5 Refining Histological Subtyping for Risk Stratification

AI may support automation and refinement of complex histological classifications. The Laurén classification has interobserver variability, and WSI‐trained DL models have been explored for automated classification and survival stratification [13]. Claims regarding AI‐based prediction of ACRG molecular subtypes should be supported by primary AI studies; otherwise, molecular classification studies using immunohistochemistry should be described as validation or reproduction of subtype frameworks rather than AI subtype prediction.

## 6 AI in Guiding Therapeutic Decisions for Precision Oncology

Given the molecular heterogeneity of GC, identifying patients who are likely to benefit from systemic therapy is a major goal of precision oncology. Several multimodal AI models have reported improved predictive performance compared with single‐modality models in selected retrospective datasets, but current evidence is insufficient to conclude consistent superiority across clinical settings [17, 61](Figure 6). Treatment‐response prediction models should therefore be interpreted as investigational tools unless validated prospectively with clinically meaningful endpoints. Representative AI applications for predicting therapeutic response in GC are summarized in Table 6.

**FIGURE 6:**
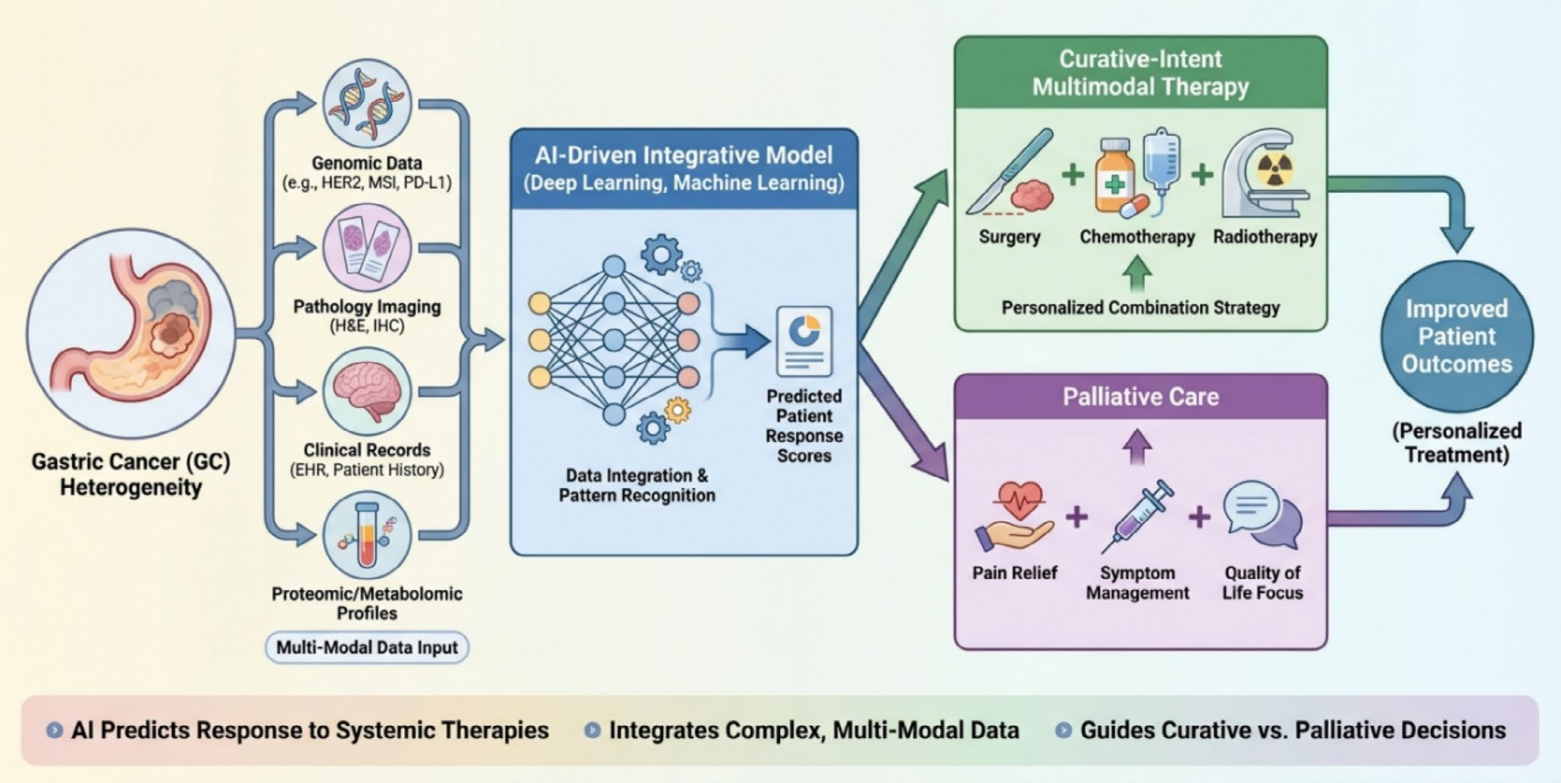
AI‐driven integrative models for therapeutic decision support in precision oncology. Given the molecular and clinical heterogeneity of gastric cancer, AI models can integrate multimodal inputs to estimate systemic therapy response and support multidisciplinary treatment planning, while final treatment decisions remain clinically governed. *Source:* Created by the authors based on the reviewed literature; no previously published figure was reused.

**TABLE 6:** Summary of AI applications in predicting therapeutic response in gastric cancer.

Name/approach	Application/function	References	Evidence appraisal/limitations
Multi‐modal AI models	Integrate diverse data (e.g., radiomics, pathomics, genomics) to predict therapy response; improved performance has been reported in selected retrospective datasets.	[17, 61]	Usually retrospective; relative multimodal performance should be assessed study by study and should not be generalized without head‐to‐head prospective evidence.
Combined clinical‐radiomic models	Combine clinical and radiomic variables for outcome prediction; selected studies report improved discrimination compared with radiomics‐only models.	[17]	Research‐stage evidence; external validation, calibration, decision‐curve analysis, and workflow utility remain limited unless explicitly reported.
MSI‐H tumor stratification	Identifies a patient subgroup with a good prognosis with surgery alone that may not benefit from neoadjuvant chemotherapy, guiding treatment decisions.	[62]	Primary clinical trial evidence is required for chemotherapy‐benefit claims; confirmatory molecular testing remains necessary.
DL radiomics nomogram	Integrates deep learning features (from CECT images via EfficientNet V2), radiomics, and clinical factors to predict neoadjuvant chemotherapy response. Outperformed single‐data‐type models with an AUC of 0.751 in external validation.	[63]	Research‐stage evidence; external validation, calibration, decision‐curve analysis, and workflow utility remain limited unless explicitly reported.

### 6.1 Predicting Response to Chemotherapy

#### 6.1.1 Neoadjuvant Chemotherapy (NAC)

Accurately predicting NAC response is clinically important because ineffective preoperative therapy may delay definitive surgery in resistant tumors. The potential lack of benefit, or possible adverse association, of perioperative chemotherapy in dMMR/MSI‐H gastroesophageal cancer should be supported by primary clinical evidence such as the exploratory MAGIC trial analysis rather than by unrelated molecular classification studies [64]. Multicenter DL models using multi‐phase CECT have reported external validation for NAC response prediction (AUC 0.751), but this level of performance does not yet establish readiness for treatment selection without prospective testing [63].

#### 6.1.2 Adjuvant Chemotherapy and Chemoresistance

In the adjuvant setting, multitask DL models (e.g., AER‐SwinT) incorporate intermediate predictions of the tumor microenvironment, such as ImmunoScore and periostin expression, to improve chemotherapy‐response modeling in selected datasets [65]. AI‐based analyses are also being used to explore molecular mechanisms of chemoresistance; for example, ML has identified PAK6 as a candidate contributor to oxaliplatin resistance via the homologous‐recombination repair pathway [66]. Longitudinal liquid‐biopsy features, including dynamic changes in circulating tumor cells analyzed by attention‐aware models, have been proposed as non‐invasive markers for monitoring therapeutic efficacy [67]. WSI‐based pathomics approaches [61] and logistic‐regression nomograms using accessible clinical factors [68] have reported promising performance. For specific histological subtypes such as signet‐ring cell carcinoma, DL‐based models have been explored for predicting potential benefit from postoperative chemotherapy [69].

#### 6.1.3 Guiding Therapy in Early GC

Predicting LNM in early‐stage (T1) GC is clinically important because it informs the choice between organ‐sparing endoscopic resection and radical gastrectomy [70]. ML algorithms applied to SEER data [71], models incorporating standardized colorimetric endoscopic images [72], and radiomics models using preoperative variables [73] have been proposed as non‐invasive risk‐stratification approaches. Combined cfDNA and CT‐based approaches also show promise for identifying selected patients in whom radical gastrectomy might be avoided, but prospective clinical validation is required [31].

### 6.2 Predicting Response to Immunotherapy

The efficacy of immune checkpoint inhibitors (ICIs) is enriched in molecularly defined subgroups such as MSI‐H/dMMR, EBV‐positive, high tumor mutational burden, or PD‐L1‐positive GC. AI models may provide a low‐cost pre‐screening approach by predicting immune‐relevant features from H&E slides or CT images, but confirmatory molecular testing remains necessary for clinical decision‐making.

#### 6.2.1 Histopathology and Radiomics

DL models analyzing WSIs have been investigated for detecting immunotherapy‐sensitive subgroups, including EBV‐positive and MSI/dMMR tumors [12, 74, 75]. These approaches should be interpreted as screening or preselection tools rather than replacements for validated molecular assays. On the radiologic front, CT‐based radiomics and DECT‐derived nomograms have been explored for preoperative MSI prediction and immune microenvironment inference [76, 77, 78], but prospective validation and clinical utility remain uncertain.

#### 6.2.2 Multi‐Omics and TME Profiling

By integrating multi‐omics data, AI models have constructed candidate cell‐death indices and ferroptosis scores. Low ferroptosis scores have been associated with an immune‐inflamed tumor microenvironment, including higher tumor mutational burden and PD‐L1 expression, suggesting potential enrichment for ICI responsiveness [79, 80, 81]. Multi‐omics signatures covering programmed cell‐death pathways (GMPS) have also been proposed for ICI‐response prediction [82]. Somatic mutations in homologous‐recombination (HR) genes have been reported to correlate with immunotherapeutic benefit in selected analyses [83, 84]. Network‐based analyses have proposed ADAMDEC1 as a candidate marker associated with ICI response, but this finding should be interpreted cautiously until confirmed in primary gastric‐cancer immunotherapy cohorts [85]. Integration of spatial and transcriptomic profiles may help identify candidate responders within microsatellite‐stable populations, but clinical utility remains unproven [74, 86, 87, 88].

### 6.3 Predicting Response to Targeted Therapies

AI may eventually support matching of targeted therapies to molecularly vulnerable tumor phenotypes, but most evidence remains exploratory. For PARP inhibitors, simple BRCA1/2 mutational status may be insufficient, and ML‐derived genomic instability or homologous recombination deficiency signatures have been proposed [89, 90, 91]. For HER2, DECT‐based radiomic nomograms have been explored as non‐invasive predictors [92], but trastuzumab eligibility still requires validated clinical biomarker testing. AI‐based prediction of CLDN18.2 positivity from routine pathology slides remains an emerging hypothesis and should be supported by primary gastric cancer studies before being framed as a demonstrated capability.

For anti‐angiogenic agents such as ramucirumab, pre‐treatment molecular classification has been explored as a possible approach to identifying subgroups that may benefit from therapy [93]. DECT‐based radiomic nomograms have been investigated as non‐invasive adjuncts for estimating HER2 status, but trastuzumab selection still requires validated biomarker testing [92]. AI may also generate hypotheses about targeted vulnerabilities; for example, diffuse GC with E‐cadherin (CDH1) loss has been linked to ferroptosis‐related therapeutic hypotheses [94]. Models predicting functional E‐cadherin loss or CLDN18.2 positivity from pathology slides remain future‐oriented tools that require primary gastric cancer validation before clinical trial enrollment can rely on them [61]. These computational predictions may be complemented by ex vivo drug screening using patient‐derived organoids (PDOs), which remain experimental platforms for personalized therapy testing [95, 96]. AI‐based prognostic models have also been explored for complex clinical scenarios, such as GC with liver metastasis [97].

### 6.4 Predicting Response to Radiotherapy

Direct evidence for AI‐guided radiotherapy response prediction in gastric cancer remains limited. Evidence from related gastrointestinal malignancies, including esophageal squamous cell carcinoma, should be interpreted as hypothesis‐generating rather than gastric cancer‐specific. This evidence gap highlights the need for GC‐specific radiotherapy cohorts, standardized endpoints, and prospective validation before AI‐based radiotherapy personalization can be discussed as clinically actionable.

## 7 Comparative Evidence Synthesis Across Modalities

Across modalities, the strongest near‐term translational evidence appears to come from image‐based AI tasks with clearly defined inputs and endpoints, such as endoscopic lesion detection, WSI‐based biomarker pre‐screening, and CT‐based staging or response prediction. Even in these areas, however, many studies remain limited by retrospective design, selected datasets, lack of prospective workflow assessment, and insufficient reporting of calibration and clinical utility.

Endoscopy‐based AI is attractive because it can operate in real time and directly affect lesion detection and characterization, but deployment depends on video‐level validation, hardware compatibility, training of endoscopists, patient‐level outcomes, and performance in nonexpert or low‐resource settings. Computational pathology models benefit from standardized digital slides and abundant morphologic information, but require robust external validation across scanners, staining protocols, and institutions. Radiomics models offer non‐invasive assessment but are sensitive to acquisition protocols, segmentation variability, feature instability, and population shift.

By contrast, omics‐based signatures, liquid‐biopsy AI, microbiome models, and treatment‐response prediction tools are generally less mature. These approaches often rely on small cohorts, public databases, retrospective endpoints, or non‐standardized assays. Their clinical value will depend on whether they add actionable information beyond established clinicopathologic variables and guideline‐approved biomarkers. Therefore, future studies should report not only AUC and accuracy, but also sensitivity, specificity, calibration, decision‐curve analysis, net benefit, external validation, and evidence of workflow impact.

## 8 Challenges, Ethical Considerations, and Future Directions

Although AI in GC management has substantial research potential, translation from model development to routine care faces technical, clinical, regulatory, and ethical barriers. These barriers are not peripheral considerations; they determine whether promising retrospective performance can become safe and useful clinical decision support (Figure 7). The major technical and clinical challenges associated with applying AI to GC are summarized in Table 7.

**FIGURE 7:**
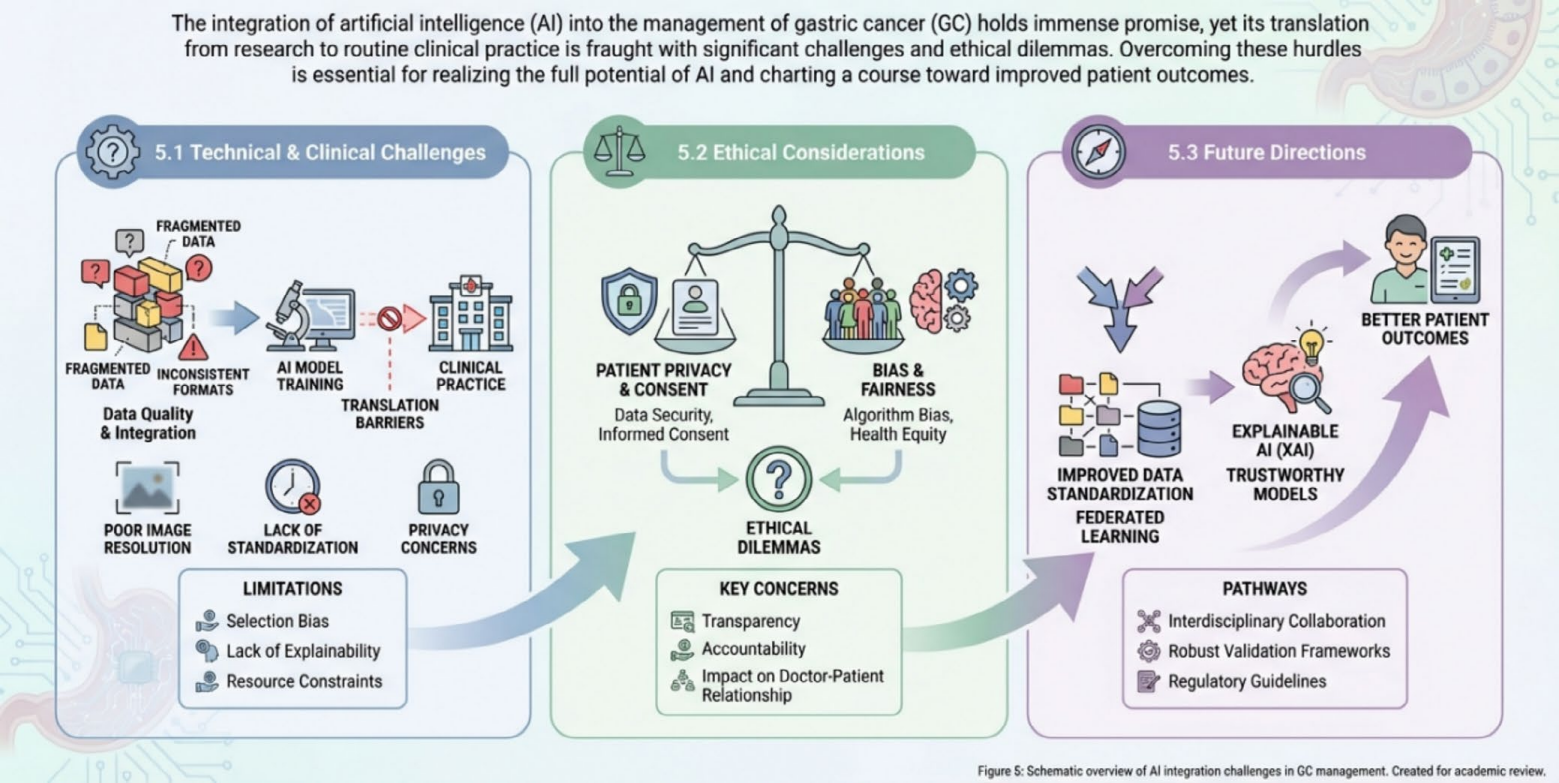
Schematic overview of technical challenges, ethical considerations, and future translational pathways for integrating AI into gastric cancer management. Moving AI from research to routine clinical practice requires attention to data fragmentation, algorithmic bias, privacy‐preserving collaboration, explainability, regulatory planning, reimbursement, and post‐deployment monitoring. *Source:* Created by the authors based on the reviewed literature; no previously published figure was reused.

**TABLE 7:** Summary of technical and clinical challenges in applying AI to gastric cancer.

Name/approach	Application/function	References	Evidence appraisal/limitations
Data quality and standardization	Insufficient data standardization and weak clinical validation are primary obstacles to the widespread adoption of AI.	[98]	A cross‐cutting translational barrier; requires standardized imaging/pathology protocols, annotation procedures, and reporting standards.
Limited generalizability	Models trained on single‐center, retrospective data often fail to generalize to different clinical settings, equipment, and patient demographics.	[24, 71, 99]	Domain shift across scanners, staining protocols, centers, and populations requires independent external and prospective validation.
Lack of real‐world validation	A major gap exists as most AI studies do not progress beyond initial retrospective assessments to prospective, real‐world validation.	[98, 99, 100]	Real‐world evidence remains limited; validation should focus on GC‐specific clinical cohorts and prospective workflow studies.
Inconsistent AI tools	Significant variability between different commercial AI solutions for the same task can lead to inconsistent clinical results and decisions.	[100]	Commercial and local tool variability requires benchmarking against common datasets, harmonized endpoints, and monitoring after deployment.
Biological heterogeneity	The inherent biological diversity of gastric cancer, including challenging subtypes like gastric signet ring cell carcinoma (GSRC), complicates AI model development.	[101, 102]	Biological heterogeneity requires GC‐specific, subtype‐aware cohorts and validation across relevant populations and assays.
Etiological complexity	Virulence factors from pathogens like *H. pylori* (e.g., oncoproteins CagA, VacA) contribute to GC heterogeneity by dysregulating host cell signaling.	[102]	Pathogen and host factors should be treated as sources of heterogeneity; model development requires population‐aware validation.
Population‐specific differences	AI models may fail to generalize across ethnicities due to distinct molecular landscapes, such as the high rate of EBV‐associated GC in the Alaska Native population.	[101, 103]	Potential algorithmic bias and underrepresentation require subgroup reporting and validation in geographically diverse cohorts.
Geographic pathogen variation	Geographic variations in pathogen oncoproteins, such as the *H. pylori* CagA motifs (EPIYA‐D vs. EPIYA‐C), influence cancer risk and challenge the creation of globally applicable models.	[102]	Geographic pathogen variation may contribute to domain shift and should be considered in globally generalizable model design.

### 8.1 Technical Bottlenecks and Data Heterogeneity

The primary impediment to AI translation is limited generalizability caused by data heterogeneity, data siloing, and inconsistent model validation [98, 99, 100]. Many current models are developed using retrospective single‐center datasets and internal validation. Models optimized for specific scanner protocols, staining workflows, population structures, or annotation practices may perform poorly when externally deployed, a problem related to domain shift and model drift.

The biological heterogeneity of GC further complicates model development. Models trained predominantly on selected Western or East Asian cohorts may not generalize to underrepresented populations, such as Alaska Native patients with distinct molecular landscapes and higher rates of EBV‐associated tumors [101]. Geographic variation in *H. pylori* virulence factors, including CagA EPIYA motifs, may also influence risk patterns and model transportability [102]. These issues support the need for diverse, multiethnic, multicenter validation cohorts rather than reliance on narrow training datasets.

### 8.2 Ethical Dilemmas and the “Black Box”

Ethical deployment of AI demands rigorous mitigation of algorithmic bias. If AI tools are disproportionately trained on data from specific ethnicities, they risk exacerbating existing health disparities [103, 104].

The opaque, “black‐box” nature of deep neural networks poses a substantial barrier to clinical trust. Addressing this necessitates the widespread adoption of Explainable AI (XAI) [105]. Techniques such as SHAP and Grad‐CAM are essential to deconstruct model outputs, ensuring that AI predictions align with established clinico‐pathological knowledge and keeping the ultimate diagnostic responsibility firmly with the attending physician [106]. Additionally, utilizing massive patient datasets necessitates stringent adherence to data privacy regulations and informed consent protocols.

### 8.3 Future Directions and the Path to Clinical Implementation

Future research should move beyond retrospective discrimination metrics toward prospective, multicenter evaluation of clinical utility, safety, cost‐effectiveness, and workflow impact. Studies should report calibration, decision‐curve analysis, subgroup performance, failure modes, and external validation results. Regulatory approval pathways, reimbursement models, medicolegal responsibility, clinician‐AI interaction, infrastructure requirements, post‐deployment monitoring, and model drift management should be considered early in model design rather than after technical development.

To overcome data siloing while protecting privacy, federated learning and secure multicenter model evaluation are promising directions. Multimodal foundation models, vision‐language models, and weakly supervised learning may reduce annotation burdens and improve cross‐task transfer, but GC‐specific evidence remains limited and should be framed as emerging rather than established. AI‐driven drug discovery and digital pathology studies should be integrated into the review as future‐oriented evidence, including recently published work recommended by the reviewers [75, 107].

For clinical integration, AI systems must be embedded into existing endoscopy, radiology, pathology, oncology, and electronic health record workflows with user‐centered interfaces. Clinicians generally need interpretable, actionable information rather than opaque risk scores. Well‐designed dashboards should clarify modifiable risk factors, uncertainty, and recommended next steps while minimizing alert fatigue [108].

## 9 Conclusion

AI has shown substantial research potential in GC diagnosis, staging, prognostication, and treatment‐response prediction, but most models remain insufficiently validated for routine clinical use. Current evidence is strongest for selected image‐based tasks such as endoscopic lesion detection, WSI‐based biomarker screening, and CT radiomics‐based staging or response prediction, whereas liquid‐biopsy AI, multi‐omics signatures, radiotherapy‐response prediction, and large multimodal AI systems remain more exploratory. Across modalities, the field must move beyond high AUC values toward robust external validation, calibration, decision‐curve analysis, prospective workflow testing, and transparent reporting of limitations. Rigorous multicenter studies, standardized endpoints, explainable models, privacy‐preserving collaboration, regulatory planning, reimbursement evaluation, and post‐deployment monitoring will determine whether AI tools can ultimately improve clinical decision‐making and patient outcomes in gastric cancer.

## Author Contributions

**Chong Chen:** conceptualization, writing – original draft. **Junpeng Zhao:** methodology. **Weisong Xu:** validation. **Kang Xu:** software, visualization. **Tong Wu:** writing – review and editing. **Xuebing Xu:** writing – review and editing. **Mingbing Xiao:** funding acquisition, writing – review and editing.

## Funding

This study was supported by grants from the National Natural Science Foundation of China (no. 82272624, 82471851), the Natural Science Foundation of Jiangsu Province (no. BK20251835), the Department of Scientific Research Development under the Ministry of Education (no. 2025XH033), the Nantong Science and Technology Bureau (Special Project for Prospective Technology Innovation, no. GZ2024007, JCZ2025009), the Nantong Municipal Talent Work Leading Group Office (2025–6‐2), and the Jiangsu Provincial Department of Education (KYCX23_3419, KYCX24_3593, KYCX25_3797, KYCX25_3789).

## Ethics Statement

The authors have nothing to report.

## Conflicts of Interest

The authors declare no conflicts of interest.

## Data Availability

No original datasets were generated or analyzed in this review. All data discussed are derived from cited published studies.
